# Linking Food Insecurity and Coping Strategies to Children’s Dietary Intake in Urban Areas in Surabaya, Indonesia

**DOI:** 10.3390/nu18162724

**Published:** 2026-08-20

**Authors:** Emyr Reisha Isaura, Aprilia Kusumawardhani, Annis Catur Adi, Charles Frans, Gloriana Seran, Sherly Vantono, Laili Rahmawati, Andianto Indrawan Tjiptohardjo, Maria Natalia Indawati, Taufiqurrahman Taufiqurrahman, Roselynne Anggraini, Yuyun Erlina Susanti, Romyun Alvy Khoiriyah

**Affiliations:** 1 Department of Nutrition, Faculty of Public Health, Universitas Airlangga, Surabaya 60115, Indonesia; 2 Department of Program, Wahana Visi Indonesia—Operational Office Surabaya, Surabaya 60238, Indonesia; charles_frans@wvi.org; 3 Department of Technical Sectors, Wahana Visi Indonesia—National Office Jakarta, Jakarta 10340, Indonesia; 4 Departement of Strategy, Risk, Innovation and Evidence, Wahana Visi Indonesia—National Office Jakarta, Jakarta 10340, Indonesia; 5 Fakultas Psikologi dan Kesehatan, Universitas Islam Negeri Sunan Ampel, Surabaya 60237, Indonesia; 6 Departement of Obstetrics and Gynecology, Faculty of Medicine, Ciputra University, Surabaya 60271, Indonesia; 7 Departement of Pediatrics, Faculty of Medicine, Ciputra University, Surabaya 60271, Indonesia; 8 Department of Nutrition, Politeknik Kesehatan Kementrian Kesehatan, Surabaya 60282, Indonesia; 9 Department of Nutrition, Faculty of Sports and Health Science, Universitas Negeri Surabaya, Surabaya 60231, Indonesia; 10 Asosiasi Dietisien Indonesia—Korwil Surabaya, Surabaya 60286, Indonesia

**Keywords:** household food insecurity, coping strategies, children’s dietary intake

## Abstract

**Background:** Food insecurity continues to pose a significant public health challenge in urban low-income communities, where limited resources and unstable food systems may negatively relate to children’s nutrition. Although prior studies have linked food insecurity with inadequate dietary intake, the role of coping strategies in this relationship remains insufficiently understood. This study investigated the associations between household food insecurity, coping strategies, and children’s dietary intake in densely populated urban areas of Surabaya, Indonesia. **Methods:** A cross-sectional study was conducted among 55 mother–child pairs in Simolawang and Bulak Banteng from September 2025 to February 2026. We used the Household Food Insecurity Access Scale (HFIAS) and the Short Food Literacy Questionnaire (SFLQ), and coping behaviors were assessed using the Reduced Coping Strategy Index (r-CSI). Children’s dietary intake was obtained through a Semi-Quantitative Food Frequency Questionnaire (SQ-FFQ). Pearson correlation and multivariate regression analyses were applied to examine relationships among variables. **Results:** Most households were classified as food secure (61.82%), although varying degrees of food insecurity were observed. Household food insecurity was not significantly associated with children’s energy or macronutrient intake. In contrast, coping strategy scores were positively associated with household food insecurity (β = 0.26–0.27), indicating greater use of coping strategies among households experiencing greater food insecurity. **Conclusions:** Food insecurity was linked to increased coping behaviors but not directly to children’s macronutrient intake. Coping mechanisms may help sustain short-term intake but do not guarantee adequate nutrition. Interventions should address both food access and dietary quality in urban poor settings.

## 1. Introduction

Food insecurity remains a critical global public health issue, particularly in low- and upper-middle-income countries undergoing rapid urbanization and socioeconomic transitions. It is defined as limited or uncertain access to sufficient, safe, and nutritious food necessary for an active and healthy life [1,2]. Despite global progress in poverty reduction, disparities in food access and dietary quality persist, especially in urban poor populations where structural inequalities, informal employment, and rising food prices exacerbate vulnerability [3,4]. In Indonesia, food insecurity continues to be associated with a substantial proportion of households, with urban settings increasingly recognized as high-risk environments due to population density, unstable income sources, and changing food systems [5,6,7]. Children are among the most vulnerable groups affected by food insecurity, as inadequate dietary intake during early life can have lasting consequences on growth, cognitive development, and overall health [8,9,10,11,12]. Previous studies demonstrated that household food insecurity was considerably associated with adverse nutritional outcomes, including stunting, wasting, and micronutrient deficiencies [13,14,15,16,17]. In Indonesia, national and regional studies have consistently reported that children living in food-insecure households are more likely to experience inadequate dietary intake and poor nutritional status compared with their food-secure counterparts [13,14,17,18,19]. Households facing food insecurity often adopt a range of coping strategies to manage limited resources. These behaviors include reducing meal frequency, decreasing portion sizes, substituting preferred foods with cheaper alternatives, relying on friends and family for food or aid (i.e., money), and relying on social networks [20,21,22,23,24].

Further, in urban Indonesian contexts, coping strategies are shaped by complex socioeconomic and environmental conditions, including limited access to social aid, fluctuating food prices, and constrained living environments. Previous studies have demonstrated that higher coping strategy index (CSI) scores are associated with poorer food security status, reduced dietary diversity, and lower nutrient adequacy [25,26,27]. Moreover, coping strategies can be directly associated with children’s food intake by altering intra-household food allocation, meal composition, and feeding practices [23,28,29,30]. These dynamics often result in increased reliance on energy-dense but nutrient-poor foods, thereby contributing to both undernutrition and emerging diet-related non-communicable diseases [31]. Despite the growing literature, there remains a limited understanding of how food insecurity, coping strategies, and children’s dietary intake interact at the local level in urban poor communities. Many studies have examined these factors independently, limiting insights into their interconnected pathways [32,33]. Furthermore, context-specific evidence is essential, as household coping behaviors and dietary patterns are correlated with local socioeconomic and cultural conditions. Therefore, the primary aim of this study was to investigate the associations between household food insecurity, coping strategies, and children’s dietary intake among urban poor households in densely populated areas (e.g., Simolawang and Bulak Banteng) of Surabaya, Indonesia. In addition, this study sought to describe household food insecurity, coping strategy use, food literacy, parenting style, and children’s dietary intake; examine the relationship between household food insecurity and coping strategies; evaluate the associations between household food insecurity and children’s dietary intake; and determine whether coping strategies were associated with children’s dietary intake independent of household food insecurity. Food literacy and parenting style were included as household characteristics to provide contextual information regarding family environments that may correlate with food-related behaviours and children’s nutrition.

The remainder of this paper is organized as follows. Section 2 describes the study design, participant recruitment, measurement instruments, data collection procedures, and statistical analyses. Section 3 presents the participants’ characteristics and the findings on household food insecurity, coping strategies, food literacy, parenting style, and children’s dietary intake, followed by the correlation and regression analyses. Section 4 discusses the findings in relation to previous studies, highlights their implications for food insecurity and child nutrition in urban poor settings, and addresses the study’s strengths and limitations. Finally, Section 5 summarizes the main conclusions and provides recommendations for future research and public health practice.

Despite growing evidence linking household food insecurity with poor child nutrition, three important knowledge gaps remain. First, relatively few studies have simultaneously examined household food insecurity, coping strategies, and children’s dietary intake within a single analytical framework. Second, evidence from densely populated urban poor communities in Indonesia is limited, despite their distinct socioeconomic and food environments. Third, little is known about whether coping strategies may help explain the apparent disconnect between household food insecurity and children’s dietary intake in these settings. Addressing these gaps is important for developing interventions that strengthen household resilience while improving children’s nutritional outcomes.

## 2. Materials and Methods

### 2.1. Study Design and Participant Characteristics

A cross-sectional study was conducted between September 2025 and February 2026 among mother–child pairs residing in Simolawang and Bulak Banteng, Surabaya, Indonesia. Participants were recruited using a snowball sampling approach after obtaining a list of eligible mothers from the local Primary Health Care Centers (PUSKESMAS). The PUSKESMAS list was used only to identify eligible participants. WhatsApp invitations were sent to these mothers, and snowball sampling was used only to identify additional eligible mothers who were not on the original list. Eligible participants were mothers aged 18 years or older who had at least one child aged 6–36 months and had resided in the study area for at least six months. Mothers of children with chronic diseases or congenital conditions known to affect dietary intake or nutritional status were excluded. Written informed consent was obtained from all participants before data collection. Sociodemographic information collected included maternal and paternal age, education, occupation, household income, food expenditure, household size, number of children, number of toddlers, and housing characteristics. Housing characteristics included building area (m^2^), floor area (m^2^), number of rooms, and household occupancy, which were collected to characterize the household living environment.

Simolawang and Bulak Banteng are densely populated urban communities in Surabaya characterized by high levels of socioeconomic vulnerability, informal employment, limited household resources, and dependence on purchased foods. These communities have been identified by local health authorities as priority areas for nutrition and food security interventions because of their relatively high prevalence of child malnutrition and household economic insecurity. Unlike rural households that may partially rely on subsistence agriculture, urban poor households in these communities depend almost entirely on local food markets, making them particularly vulnerable to fluctuations in food prices and household income. Consequently, these settings provide an important context for examining how food insecurity is associated with household coping behaviors and children’s dietary intake. Although these communities are not intended to represent all Indonesian households, they reflect many characteristics commonly observed among low-income urban settlements in large Indonesian cities, including high population density, unstable employment, and dependence on purchased foods.

Anthropometric measurements of the children were conducted through direct, face-to-face assessments at designated community or health service centers. Measurements included body weight and height. Height and weight were measured using calibrated digital instruments. Children’s body weight was measured using a calibrated digital weighing scale (OneMed OD 231B, Zhongshan Camry Electronic Co., Ltd., Zhongshan, China), accurate to 0.1 kg, while recumbent length or standing height (according to age) was measured using a portable length board or stadiometer (OneHealth OH-JH05, PT Era Medika Alkesindo, Medan, Indonesia), accurate to 0.1 cm if the children were aged >24 months. All anthropometric measurements were performed by trained research staff following WHO-standardized measurement procedures. Each measurement was obtained under standardized conditions with children wearing light clothing and no shoes. Age- and sex-specific anthropometric indices were calculated from the measured anthropometric data. Weight-for-age z-score (WAZ), height-for-age z-score (HAZ), and weight-for-height z-score (WHZ) were derived using the WHO Child Growth Standards. These z-scores were used to describe children’s anthropometric status in relation to the WHO reference population. The anthropometric indicators were presented descriptively and were not included as primary outcomes in the regression analyses.

Sociodemographic information collected included parental age, education level, occupation, household income, household food expenditure, household size, and housing characteristics. In addition, food literacy and parenting style were assessed because both have been shown to be associated with household food purchasing decisions, food preparation practices, child feeding behaviors, and ultimately children’s dietary intake, particularly among socioeconomically disadvantaged families. Food literacy was assessed using the Short Food Literacy Questionnaire (SFLQ), a validated instrument developed to evaluate individuals’ knowledge and competencies related to food planning, food selection, food preparation, nutrition information appraisal, and healthy eating behaviors. The questionnaire consists of 12 items rated using a Likert-type response scale, with higher total scores indicating better food literacy. Previous studies have demonstrated satisfactory validity and internal consistency (Cronbach’s α: 0.891) across adult populations [34]. The Indonesian version has also been used in community-based nutrition research with acceptable psychometric properties.

Parenting style was assessed using the Parenting Styles and Dimensions Questionnaire (PSDQ), which classifies parenting practices into three dominant styles: authoritative, authoritarian, and permissive. The questionnaire evaluates parents’ behavioral approaches toward child discipline, responsiveness, communication, and autonomy support. Scores for each parenting style were calculated according to the instrument guidelines, with higher scores representing stronger endorsement of that parenting style. The PSDQ has demonstrated good construct validity and reliability in numerous countries, including adaptations used among Indonesian parents (Cronbach’s α: 0.86) [35].

### 2.2. Food Insecurity, Coping Strategy, and Dietary Intake Assessment

Household food insecurity was assessed using the Household Food Insecurity Access Scale (HFIAS) developed by the Food and Nutrition Technical Assistance (FANTA) Project. The HFIAS is a widely validated instrument designed to measure the access dimension of household food insecurity over the previous four weeks. It comprises nine occurrence questions covering three domains: (1) anxiety and uncertainty regarding household food supply, (2) insufficient food quality, and (3) insufficient food quantity and its physical consequences [36,37]. Each occurrence question is followed by a frequency-of-occurrence response, generating a total score ranging from 0 to 27, where higher scores indicate greater household food insecurity. The HFIAS has demonstrated good validity and reliability across low- and middle-income countries and has previously been applied in Indonesian populations [37].

On the other hand, household coping behaviors were evaluated using the Reduced Coping Strategy Index (rCSI), a standardized indicator developed by the World Food Programme to quantify behavioral responses adopted when households experience food shortages. The rCSI measures the frequency of five food-related coping strategies during the previous seven days: relying on less preferred foods, borrowing food or money to purchase food, limiting portion sizes, restricting adults’ food consumption for children, and reducing the number of daily meals. Responses are weighted according to the standardized rCSI scoring system, resulting in total scores ranging from 0 to 56, with higher scores indicating greater reliance on food-related coping strategies and increased food insecurity. The rCSI has been extensively validated internationally and is widely used for food security monitoring in low-resource settings [38].

Moreover, children’s habitual dietary intake during the previous month was assessed using a semi-quantitative food frequency questionnaire (SQ-FFQ). Mothers reported the frequency and portion sizes of foods and beverages consumed by their children. Daily nutrient intakes, including energy, carbohydrate, protein, and fat, were calculated using NutriSurvey for Windows (Version 2007, EBISpro, Stuttgart, Germany) [39] and compared with the Indonesian Recommended Dietary Allowances (Angka Kecukupan Gizi, AKG) issued by the Ministry of Health of the Republic of Indonesia according to the child’s age and sex. Further, nutrient intake values were calculated primarily to describe dietary intake patterns and were compared with the age- and sex-specific Indonesian RDA for descriptive purposes only. The regression analyses were performed using continuous nutrient intake variables rather than adequacy classifications. Energy intake was estimated from the SQ-FFQ and presented as absolute daily intake (kcal/day). Individual energy requirements were not estimated because detailed physical activity data were not collected. Therefore, energy intake values were interpreted descriptively rather than as indicators of adequacy. All questionnaires used in this study were selected because they have been previously validated for measuring their respective constructs in nutritional and public health research. Standardized administration procedures were followed throughout data collection to ensure consistency. Where available, Indonesian versions with established psychometric properties were used.

### 2.3. Conceptual Framework and Variable Selection

Variable selection was guided by the conceptual framework developed from previous studies on household food insecurity and child nutrition. Household food insecurity (HFIAS score) was considered the primary exposure because it represents the household’s ability to access sufficient food. Household coping strategies (rCSI score) were considered an intermediate behavioral response to food insecurity, while children’s dietary intake (energy, carbohydrate, protein, and fat intake) represented the primary nutritional outcomes. Food literacy, parenting style, household income, household size, and parental education were measured as contextual household characteristics that may correlate with food acquisition, food preparation, and child feeding practices. Given the exploratory nature of the study and the relatively small sample size, these contextual variables were primarily described descriptively and considered during model development but were not included in the final adjusted regression models.

### 2.4. Statistical Analysis

Data were analyzed using Stata version 17.0 (StataCorp LLC, College Station, TX, USA). Descriptive statistics were used to summarize participants’ characteristics. Categorical variables were presented as frequencies and percentages, whereas continuous variables were summarized using means and standard deviations (SD). The normality of continuous variables was assessed using the Shapiro–Wilk test before inferential analyses. Variables with approximately normal distributions were analyzed using parametric methods. Pearson correlation analysis was performed to examine the bivariate associations between household food insecurity, coping strategies, and children’s dietary intake. Correlation coefficients (r) were interpreted according to the strength and direction of the linear relationships among continuous variables. Continuous variables were compared between boys and girls using the independent *t*-test, and categorical variables were compared using the chi-square test, as appropriate. All tests were two-sided, and a *p*-value < 0.05 was considered statistically significant.

Multivariable linear regression analyses were subsequently conducted because the primary outcome variables (energy intake, carbohydrate intake, protein intake, fat intake, and rCSI score) were continuous variables with approximately normal distributions. Separate regression models were fitted for each outcome variable. Household food insecurity, measured using the Household Food Insecurity Access Scale (HFIAS) total score, was specified as the primary independent variable based on the study’s conceptual framework, which hypothesized that increasing food insecurity would be related to household coping behaviors and children’s dietary intake. The Reduced Coping Strategy Index (rCSI) score was included as both an outcome variable (when examining its association with household food insecurity) and as an explanatory variable (when evaluating its association with children’s dietary intake), reflecting its proposed role as a behavioral response to household food insecurity.

Two regression models were estimated. Model 1 was an unadjusted model that examined the crude association between the exposure and outcome variables. Model 2 adjusted for children’s age (months) and sex because these variables are well-established determinants of children’s dietary intake and nutritional requirements. Other household variables, including household size, number of children, parental education, food literacy, parenting style, and household income, were considered potential confounders during model development. However, they were not retained in the final adjusted models because of the limited sample size (*n* = 55), which could increase the risk of model overfitting and unstable coefficient estimates.

Household food security categories were derived from the standard HFIAS classification guidelines, with households categorized as food secure, mildly food insecure, moderately food insecure, or severely food insecure based on the frequency and severity of affirmative responses. Similarly, rCSI scores were categorized into low, medium, and high coping strategy groups using the standard Reduced Coping Strategy Index classification to facilitate descriptive presentation. However, regression analyses used the continuous HFIAS and rCSI scores to maximize statistical power and preserve information. Statistical significance was defined as a two-sided *p* value < 0.05.

## 3. Results

### 3.1. Participant Characteristics

Table 1 shows an overview of qualitative variables on participant demographics of 55 participants, with more boys (60.00%) than girls (40.00%). Parents had low education levels (96.36%). Mothers were housewives (80.00%), while fathers worked as laborers (52.73%). Over half of the households were food secure (61.82%), though some experienced mild to severe food insecurity. The families had low coping strategy levels (69.09%). Food literacy was mainly moderate (40.00%), and the common parenting style was authoritarian (60.00%).

On the other hand, Table 2 shows that most measured characteristics—anthropometric indices, parental age, household composition, income, food expenditure, dietary intake, and food security—were broadly similar between boys and girls, with no statistically significant differences (*p* > 0.05). Children generally had negative z-scores for weight and height, indicating suboptimal growth overall. Dietary intake and food variety were comparable across groups. However, a few differences emerged: girls had significantly higher egg protein source scores, while boys had significantly higher total food literacy scores and higher Reduced Coping Strategy Index (CSI) scores. Overall, the key message is that sex differences were minimal across most variables, with only a small number of specific behavioral and dietary indicators showing significant variation.

### 3.2. Food Insecurity and Children’s Dietary Intake

Table 3 presents the correlation analysis, which shows that dietary intake variables are strongly interrelated, while food insecurity measures show limited association with actual nutrient intake. The Reduced Coping Strategy Index (rCSI) shows a significant positive correlation with HFIAS (r = 0.334), suggesting that greater food insecurity is associated with increased use of coping strategies. The significant positive relationships between total energy intake and macronutrients—carbohydrates (r = 0.933), protein (r = 0.942), and fat (r = 0.876)—indicate that higher energy intake is closely linked with higher consumption of all macronutrients. Significant positive correlations are also observed among macronutrients themselves, particularly between protein and fat (r = 0.899), carbohydrate and protein (r = 0.798), and carbohydrate and fat (r = 0.651). In contrast, household food insecurity (HFIAS) is not significantly associated with energy or macronutrient intake.

Table 4 presents the regression analyses, which show that household food insecurity (HFIAS) and coping strategies (rCSI) are not significantly associated with energy or macronutrient intake (carbohydrate, protein, and fat) in either unadjusted or adjusted models (all *p* > 0.05), indicating no clear relationship between food insecurity measures and actual dietary intake. However, a consistent finding across both models is that rCSI is positively and significantly associated with HFIAS (unadjusted: β = 0.27, *p* = 0.013; adjusted: β = 0.26, *p* = 0.037), suggesting that higher food insecurity is associated with greater use of coping strategies. Model 1 was an unadjusted regression model, while model 2 was adjusted for the children’s age and sex. Adjusting for children’s age and sex does not materially change these results. Overall, the key message is that food insecurity relates to coping behaviors but does not appear to directly relate to energy or macronutrient consumption in this sample.

## 4. Discussion

This study examined the interrelationship between household food insecurity, coping strategies, and children’s dietary intake in urban poor settings in Surabaya. Consistent with the study rationale outlined in the introduction, we sought to understand not only whether food insecurity was associated with dietary intake, but also how coping mechanisms may correlate with other variables in this relationship within a highly constrained urban environment. Overall, the study findings suggest that although food insecurity is clearly associated with the adoption of coping strategies, it does not appear to directly translate into measurable differences in children’s energy and macronutrient intake in this sample. The high proportion of households classified as food secure (61.82%) alongside the persistence of moderate to severe food insecurity (approximately 24%) reflects the heterogeneity typical of urban poor populations. This aligns with prior research indicating that urban settings often mask food insecurity through informal coping systems and fluctuating income streams rather than stable access to adequate nutrition [40]. Despite the relatively high food security classification, children’s anthropometric indicators (negative weight-for-age and weight-for-height z-scores) suggest underlying nutritional vulnerability. This apparent discrepancy reinforces the argument that conventional food security measures may not fully capture nutritional adequacy, particularly in dense urban environments where diet quality may be compromised despite caloric sufficiency [41,42].

A central finding of this study is the lack of significant association between household food insecurity (HFIAS) and children’s dietary intake, including energy, carbohydrate, protein, and fat [43,44]. This contrasts with a substantial body of literature reporting that food insecurity is associated with reduced dietary intake and poorer nutritional outcomes in children [17,45,46,47]. However, similar null findings have been observed in contexts where households employ adaptive coping mechanisms that are related to immediate dietary deficits [30,48,49]. In this study, the widespread use of coping strategies—although mostly categorized as low—may have played a stabilizing role in maintaining children’s caloric and macronutrient intake despite resource constraints. The strong positive correlations observed between energy and macronutrient intake (e.g., energy with protein, carbohydrate, and fat) indicate internal consistency in dietary patterns, suggesting that when children consume more energy, it is proportionally distributed across macronutrients. However, these correlations do not necessarily reflect dietary quality. The absence of association between food insecurity and these intake measures implies that households may prioritize maintaining quantity over quality, potentially relying on energy-dense but nutrient-poor foods. This interpretation is consistent with prior studies highlighting a shift toward cheaper, calorie-rich diets in food-insecure populations, which may contribute to the dual burden of malnutrition [50,51].

Importantly, the study found a consistent and statistically significant association between food insecurity (HFIAS) and the coping strategy index (rCSI) across both unadjusted and adjusted regression models. This confirms that as households experience greater food insecurity, they increasingly rely on behavioral adaptations such as reducing portion sizes, altering food choices, or borrowing food. This finding aligns with the conceptual framework presented in the introduction, where coping strategies function as an intermediate response to constrained food access. It also supports previous research indicating that CSI is a sensitive proxy indicator of food insecurity severity [50,52,53]. However, the lack of a mediating effect of coping strategies on dietary intake in this study suggests that these strategies may be effective in preserving short-term consumption levels, particularly macronutrient intake, but potentially at the expense of dietary diversity and micronutrient adequacy—variables not fully captured in the current analysis. This highlights a critical distinction between food security and nutritional security, emphasizing that maintaining caloric intake does not guarantee adequate nutrition.

Another noteworthy finding is the minimal sex difference across most variables, including dietary intake and food security indicators. Although girls had higher egg protein scores and boys had higher food literacy and coping strategy scores, these differences were limited and do not suggest systemic sex disparities in food allocation within households. This may reflect relatively equitable intra-household food distribution practices in this setting, although further investigation with larger samples would be needed to confirm this pattern. The socioeconomic profile of the sample—characterized by low parental education, informal employment (predominantly laborers), and high food expenditure-to-income ratios—provides important contextual insight. With households allocating a substantial proportion of income to food (mean ratio 0.74), financial vulnerability is evident. Yet, the ability to maintain dietary intake suggests reliance on informal support systems, market accessibility, or food substitution strategies typical of urban environments. These structural and environmental factors likely play a critical role in shaping the observed relationships and may explain why direct associations between food insecurity and intake were not detected.

Several limitations should be considered when interpreting these findings. First, the cross-sectional design limits causal inference and prevents assessment of temporal dynamics between food insecurity and dietary intake. Second, the relatively small sample size (*n* = 55) may reduce statistical power to detect subtle associations. Third, dietary intake was assessed using a SQ-FFQ, which may not fully reflect habitual intake and is subject to recall bias. Finally, the analysis focused primarily on macronutrient intake, without a detailed assessment of micronutrient adequacy or dietary quality, which are critical dimensions of child nutrition. Despite these limitations, this study contributes valuable context-specific evidence to the limited literature on urban food insecurity in Indonesia. It highlights that in dense urban settings, coping strategies may mitigate the immediate impact of food insecurity on children’s dietary intake, thereby obscuring expected associations. Future research should adopt longitudinal designs and incorporate more comprehensive dietary quality indicators to better capture the nutritional consequences of food insecurity. Additionally, interventions should move beyond food access alone and address dietary quality, resilience of coping mechanisms, and structural determinants such as income stability and food system accessibility.

## 5. Conclusions

The present findings have several implications for nutrition policy and public health practice in urban low-income communities. Although household food insecurity was not directly associated with children’s energy or macronutrient intake in this study, the significant association between food insecurity and coping strategies suggests that households adopt behavioral responses to mitigate food shortages. These coping strategies may be temporarily associated with children’s dietary intake but may not be sustainable over time. Therefore, interventions should focus not only on improving household food access but also on strengthening household resilience to food insecurity. At the community level, strengthening existing nutrition programmes, including growth monitoring, nutrition education, and complementary feeding counselling delivered through community health posts (Posyandu) and primary health care centres (Puskesmas), may help improve caregivers’ capacity to provide nutritionally adequate diets for young children. Efforts to improve access to affordable, nutrient-rich foods through social protection programmes, food assistance initiatives, local food systems, and community-based nutrition interventions should also be prioritised in densely populated urban settlements. Routine screening for household food insecurity using validated tools such as the Household Food Insecurity Access Scale (HFIAS) could be incorporated into maternal and child health services to facilitate early identification of vulnerable households and enable timely referral to nutrition and social support programmes. Integrating food insecurity screening into primary health care may improve the effectiveness of interventions targeting children at risk of inadequate dietary intake and poor nutritional outcomes.

## Figures and Tables

**Table 1:** Participants’ characteristics in the sample (*n* = 55).

Variable	Distribution
Child’s Sex	
Boy	33 (60.00)
Girl	22 (40.00)
Mother’s Education Level	
Low	53 (96.36)
High	2 (3.64)
Mother’s Work Type	
Housewife	44 (80.00)
Entrepreneur	4 (7.27)
Employee	3 (5.45)
Laborer	4 (7.27)
Father’s Education Level	
Low	53 (96.36)
High	2 (3.64)
Father’s Work Type	
Unemployment	1 (1.82)
Entrepreneur	10 (18.18)
Employee	15 (27.27)
Laborer	29 (52.73)
Household Food Insecurity Level	
Food Secure	34 (61.82)
Slightly Food Insecure	8 (14.55)
Moderately Food Insecure	5 (9.09)
Severely Food Insecure	8 (14.55)
Reduced Coping Strategy Index Categories	
Low	38 (69.09)
Medium	9 (16.36)
High	8 (14.55)
Food Literacy Level	
Low	20 (36.36)
Moderate	22 (40.00)
High	13 (23.64)
Dominant Parenting Style	
Authoritarian	33 (60.00)
Authoritative	9 (16.36)
Permissive	13 (23.64)

Note: Distribution is presented using number and percentage, or *n* (%).

**Table 2:** Anthropometrics, HFIAS, rCSI, SFLQ, and parenting scores of the sample (*n* = 55).

Variable	Total	Boy (*n* = 33)	Girl (*n* = 22)	*p*-Value
Children’s Age, month	19.09 (7.95)	18.64 (9.04)	19.77 (6.09)	0.608
Mother’s Age, year	31 (9)	30.62 (8.64)	31.84 (8.20)	0.603
Father’s Age, year	35 (7)	34.69 (6.94)	35.22 (7.53)	0.789
Weight-for-Age z-score	−2.29 (0.62)	−2.20 (0.57)	−2.42 (0.67)	0.208
Height-for-Age z-score	−0.99 (1.07)	−0.92 (1.06)	−1.13 (1.11)	0.483
Weight-for-Height z-score	−2.47 (0.77)	−2.41 (0.68)	−2.54 (0.89)	0.545
Building Area, m^2^	47.40 (47.29)	52.24 (55.54)	40.14 (30.96)	0.357
Floor Area, m^2^	37.00 (40.25)	42.55 (48.54)	28.68 (21.32)	0.214
Total Room Number	3 (2)	3.60 (2.36)	2.91 (1.72)	0.239
Total Household Members	4 (2)	4.18 (1.18)	3.82 (1.92)	0.388
Total Household Number	2 (1)	1.55 (0.79)	1.50 (0.67)	0.826
Total Individuals	6 (3)	5.58 (2.06)	5.95 (3.17)	0.593
Total Toddler Number	2 (1)	2 (1)	1 (1)	0.102
Total Family Income, Rupiahs	2,758,727 (1,209,731)	2,838,788 (1,276,809)	2,638,636 (1,119,729)	0.553
Total Food Expenditure, Rupiahs	1,909,091 (2,005,458)	2,219,697 (2,486,022)	1,443,182 (738,450)	0.162
Food Expenditure-to-Total Income Ratio	0.74 (0.71)	0.85 (0.89)	0.57 (0.22)	0.156
Energy, kcal	961.85 (423.44)	922.28 (396.00)	1021.20 (464.72)	0.401
Carbohydrate, grams	119.87 (58.45)	115.03 (56.41)	127.13 (61.99)	0.457
Protein, grams	36.69 (16.65)	35.41 (16.06)	38.60 (17.71)	0.491
Fat, grams	37.61 (18.05)	35.82 (17.40)	40.31 (19.07)	0.371
Total HFIAS Score	4.42 (6.88)	5.09 (7.49)	3.41 (5.87)	0.379
Total Reduced CSI Score	5.69 (8.40)	7.36 (9.29)	3.18 (6.22)	0.070
Total SFLQ Score	26.62 (6.00)	28.45 (5.57)	23.86 (5.67)	0.004
Total Authoritative Score	3.32 (0.59)	3.43 (0.63)	3.16 (0.51)	0.091
Total Authoritarian Score	3.66 (0.60)	3.76 (0.63)	3.53 (0.54)	0.168
Total Permissive Score	3.34 (0.64)	3.44 (0.70)	3.18 (0.50)	0.138
Connection Dimension Score (*Warmth and Support*)	3.68 (0.70)	3.8 (0.73)	3.5 (0.61)	0.119
Regulation Dimension Score (*Reasoning*/*Induction*)	3.32 (0.70)	3.43 (0.78)	3.15 (0.54)	0.155
Autonomy Granting Dimension Score (*Democratic Participation*)	2.97 (0.69)	3.07 (0.70)	2.82 (0.65)	0.179
Physical Coercion Dimension Score	3.92 (0.65)	4.00 (0.64)	3.80 (0.67)	0.259
Verbal Hostility Dimension Score	3.28 (0.80)	3.41 (0.82)	3.08 (0.74)	0.137
Non-Reasoning/Punitive Dimension Score	3.80 (0.73)	3.86 (0.81)	3.70 (0.60)	0.458
Indulgent Dimension Score	3.34 (0.64)	3.44 (0.70)	3.18 (0.50)	0.138

Note: Distribution is presented using mean and standard deviation, or mean (SD). Total room number is the number of rooms in the participants’ homes. Total household members is the total number of family members in one registered government family card or kartu keluarga. Total household number is the total number of registered government family cards for households living in one house. The total family income, total family expenditure, and expenditure-to-income ratios were calculated using the participants’ family records in one family card. We used an independent *t*-test to compare boys and girls for the continuous data and a chi-square test to compare the categorical data.

**Table 3:** Pearson correlation analysis between variables.

Dependent Variable	Independent Variable	r	*p*-Value
Energy, kcal	Carbohydrate, grams	0.9326	<0.001
	Protein, grams	0.9423	<0.001
	Fat, grams	0.8762	<0.001
	Total Score HFIAS	0.0722	0.6002
	Total Score rCSI	−0.1762	0.1981
Carbohydrate, grams	Protein, grams	0.7975	<0.001
	Fat, grams	0.6506	<0.001
	Total Score HFIAS	0.0484	0.7255
	Total Score rCSI	−0.1305	0.3422
Protein, grams	Fat, grams	0.8994	<0.001
	Total Score HFIAS	0.0997	0.4690
	Total Score rCSI	−0.1794	0.1899
Fat, grams	Total Score HFIAS	0.0757	0.5827
	Total Score rCSI	−0.2003	0.1426
Total Score HFIAS	Total Score rCSI	0.3342	0.0126
	Total score SFLQ	0.1710	0.2120
	Total Authoritative Score	0.0728	0.5975
	Total Authoritarian Score	−0.0649	0.6379
	Total Permissive Score	0.0466	0.7352
	Total Household Members	−0.0315	0.8196
	Food Expenditure-to-Total Income Ratio	−0.0772	0.5752
Total Score rCSI	Total Score SFLQ	0.0166	0.9040

**Table 4:** Regression analyses of variables.

DV	IV	Coefficient	95% CI	*p*-Value
Model 1				
Energy, kcal	Total Score HFIAS	4.45	−12.46 to 21.35	0.600
	Total Score rCSI	−8.88	−22.55 to 4.78	0.198
Carbohydrate, grams	Total Score HFIAS	0.41	−1.93 to 2.75	0.726
	Total Score rCSI	−0.91	−2.81 to 0.99	0.342
Protein, grams	Total Score HFIAS	0.24	−0.42 to 0.90	0.469
	Total Score rCSI	−0.35	−0.89 to 0.18	0.190
Fat, grams	Total Score HFIAS	0.20	−0.52 to 0.92	0.583
	Total Score rCSI	−0.43	−1.01 to 0.15	0.143
Total Score HFIAS	Total Score rCSI	0.27	0.06 to 0.49	0.013
Model 2				
Energy, kcal	Total Score HFIAS	7.69	−9.06 to 24.44	0.361
	Total Score rCSI	−2.92	−18.35 to 12.51	0.706
Carbohydrate, grams	Total Score HFIAS	0.86	−1.44 to 3.17	0.455
	Total Score rCSI	0.06	−2.06 to 2.18	0.955
Protein, grams	Total Score HFIAS	0.35	−0.31 to 1.02	0.289
	Total Score rCSI	−0.17	−0.78 to 0.44	0.583
Fat, grams	Total Score HFIAS	0.30	−0.42 to 1.03	0.406
	Total Score rCSI	−0.29	−0.96 to 0.37	0.383
Total Score HFIAS	Total Score rCSI	0.26	0.02 to 0.51	0.037

Note: Model 1 was an unadjusted regression model, while model 2 was adjusted for the children’s age and sex.

## Data Availability

The data presented in this study are available on request from the corresponding author due to ethical concerns.

## Abbreviations

The following abbreviations are used in this manuscript:

HFIAS	Household Food Insecurity Scale
rCSI	Reduced Coping Strategy Index
SQ-FFQ	Semi-Quantitative Food Frequency Questionnaire
PUSKESMAS	Pusat Kesehatan Masyarakat/Primary Health Care
FANTA	Food and Nutrition Technical Assistance Project
SFLQ	Short Food Literacy Questionnaire

## References

[1] UNICEF (2023). The State of Food Security and Nutrition in the World 2023.

[2] Rabbitt M.P., Hales L.J., Burke M.P., Coleman-Jensen A. (2023). Household Food Security in the United States in 2022.

[3] Crush J.S., Frayne G.B. (2011). Urban food insecurity and the new international food security agenda. Dev. South. Afr..

[4] Battersby J., Watson V. (2018). Urban Food Systems Governance and Poverty in African Cities.

[5] Nurhasan M., Maulana A.M., Ariesta D.L., Usfar A.A., Napitupulu L., Rouw A., Hurulean F., Hapsari A., Heatubun C.D., Ickowitz A. (2022). Toward a sustainable food system in West Papua, Indonesia: Exploring the links between dietary transition, food security, and forests. Front. Sustain. Food Syst..

[6] Khatri P., Kumar P., Shakya K.S., Kirlas M.C., Tiwari K.K. (2024). Understanding the intertwined nature of rising multiple risks in modern agriculture and food system. Environ. Dev. Sustain..

[7] Behrens P., Champagne C.M., Halford J.C., Moodie M., Proietto J., Rutter G.A., Samaras K., Holly J.M. (2025). Obesity and climate change: Co-crises with common solutions. Front. Sci..

[8] Fram M.S., Frongillo E.A., Jones S.J., Williams R.C., Burke M.P., DeLoach K.P., Blake C.E. (2011). Children are aware of food insecurity and take responsibility for managing food resources. J. Nutr..

[9] Drennen C.R., Coleman S.M., Ettinger de Cuba S., Frank D.A., Chilton M., Cook J.T., Cutts D.B., Heeren T., Casey P.H., Black M.M. (2019). Food Insecurity, Health, and Development in Children Under Age Four Years. Pediatrics.

[10] Velardo S., Pollard C.M., Shipman J., Booth S. (2021). How do disadvantaged children perceive, understand and experience household food insecurity?. Int. J. Environ. Res. Public Health.

[11] Akbari M., Foroudi P., Shahmoradi M., Padash H., Parizi Z.S., Khosravani A., Ataei P., Cuomo M.T. (2022). The evolution of food security: Where are we now, where should we go next?. Sustainability.

[12] Drewnowski A. (2022). Food insecurity has economic root causes. Nat. Food.

[13] Harper A., Rothberg A., Chirwa E., Sambu W., Mall S. (2023). Household Food Insecurity and Demographic Factors, Low Birth Weight and Stunting in Early Childhood: Findings from a Longitudinal Study in South Africa. Matern. Child Health J..

[14] Islam B., Ibrahim T.I., Wang T., Wu M., Qin J. (2025). Current trends in household food insecurity, dietary diversity, and stunting among children under five in Asia: A systematic review. J. Glob. Health.

[15] Khan Z., Ali A. (2023). Global food insecurity and its association with malnutrition. Emerg. Chall. Agric. Food Sci..

[16] Moosavian S.P., Awlqadr F.H., Mehrabani S., Mazinani K., Jalili F., MacIsaac F., Ghoreishy S.M., Ebrahimi B., Hojjati Kermani M.A., Moradi S. (2026). The Association Between Food Insecurity and Adverse Health Outcomes in Children and Adolescents: An Umbrella Review. Food Sci. Nutr..

[17] Patriota É.S., Abrantes L.C., Figueiredo A.C., Pizato N., Buccini G., Gonçalves V.S. (2024). Association between household food insecurity and stunting in children aged 0−59 months: Systematic review and meta-analysis of cohort studies. Matern. Child Nutr..

[18] Titaley C.R., Ariawan I., Iwan R.F., Tjandrarini D.H., Nazarina N., Widodo Y., Dibley M.J. (2026). Stunting in children aged under 2 years living in the eastern part of Indonesia: Analysis of the 2010–2018 Indonesia Basic Health Research. Br. J. Nutr..

[19] Beal T., Tumilowicz A., Sutrisna A., Izwardy D., Neufeld L.M. (2018). A review of child stunting determinants in Indonesia. Matern. Child Nutr..

[20] Sajwan K., Singh S.P. (2025). Multidimensionality of Household Food Security: A Systematic Review. Curr. Nutr. Rep..

[21] Ambikapathi R., Rothstein J.D., Yori P.P., Olortegui M.P., Lee G., Kosek M.N., Caulfield L.E. (2018). Food purchase patterns indicative of household food access insecurity, children’s dietary diversity and intake, and nutritional status using a newly developed and validated tool in the Peruvian Amazon. Food Secur..

[22] Mayer V.L., McDonough K., Seligman H., Mitra N., Long J.A. (2016). Food insecurity, coping strategies and glucose control in low-income patients with diabetes. Public Health Nutr..

[23] Leroy J.L., Ruel M., Frongillo E.A., Harris J., Ballard T.J. (2015). Measuring the Food Access Dimension of Food Security: A Critical Review and Mapping of Indicators. Food Nutr. Bull..

[24] Hadley C., Crooks D.L. (2012). Coping and the biosocial consequences of food insecurity in the 21st century. Am. J. Phys. Anthropol..

[25] Headey D., Hirvonen K., Alderman H. (2024). Estimating the cost and affordability of healthy diets: How much do methods matter?. Food Policy.

[26] Hirvonen K., Bai Y., Headey D., Masters W.A. (2020). Affordability of the EAT—Lancet reference diet: A global analysis. Lancet Glob. Health.

[27] Mekonen D., Bazezew A., Anteneh M., Kassie T. (2023). Analysis of food security using various indicators for policy implications: Empirical evidence from the two large cities of Bahir Dar and Gondar, the Amhara region, Ethiopia. Geo Geogr. Environ..

[28] Leroy J.L., Frongillo E.A. (2019). Perspective: What Does Stunting Really Mean? A Critical Review of the Evidence. Adv. Nutr..

[29] Asma K.M., Kotani K. (2023). Intrahousehold food intake inequality by family roles and age groups. Nutrients.

[30] Elolu S., Agako A., Okello D.M. (2023). Household food security, child dietary diversity and coping strategies among rural households. The case of Kole District in northern Uganda. Dialogues Health.

[31] Gibson E., Stacey N., Sunderland T.C., Adhuri D.S. (2021). Coping or adapting? Experiences of food and nutrition insecurity in specialised fishing households in Komodo District, eastern Indonesia. BMC Public Health.

[32] Brunet G., Fajardo G., Costa M., Bonilla L., González F., Bentancor S., Verdier S., Curutchet M.R., Girona A., Pochellú L. (2025). How do households cope with food insecurity? Quantitative analysis of the coping strategies of households with children attending public childcare in Uruguay. Appetite.

[33] Gebremichael B., Beletew B., Bimerew M., Haile D., Biadgilign S., Baye K. (2022). Magnitude of urban household food insecurity in East Africa: A systematic review and meta-analysis. Public Health Nutr..

[34] Haikal H., Suhito H.P., Duong T.V., Utami F.A., Setiono O., Puspita F.D., Rachmani E. (2024). Validation Short Food Literacy Questionnaire for Women: Translation and Cultural Adaptation in Indonesia. Int. J. Health Lit. Sci..

[35] Rahmawati A., Fajrianthi F., Purwono U. (2022). The Psychometric Properties of Parenting Styles and Dimensions Questionnaire-Short Form in Indonesia. Int. J. Eval. Res. Educ..

[36] Berhe R., Arora A., Ekanayake K., Agho K.E. (2026). Food insecurity among migrants and refugees in high-income countries: A meta-analysis of observational studies. Food Secur..

[37] Coates J., Swindale A., Bilinsky P. (2007). Household Food Insecurity Access Scale (HFIAS) for Measurement of Food Access: Indicator Guide: Version 3.

[38] Maxwell D., Caldwell R., Langworthy M. (2008). Measuring food insecurity: Can an indicator based on localized coping behaviors be used to compare across contexts?. Food Policy.

[39] Satyaningrum S.T. (2019). Perbedaan Hasil Analisis Kandungan Energi dan Makronutrien pada Formula Enteral Homemade Menggunakan Software Nutrisurvey dan Uji Laboratorium. Bachelor’s Thesis.

[40] Roy A.-S., Mazaniello-Chézol M., Rueda-Martinez M., Shafique S., Adams A.M. (2023). Food systems determinants of nutritional health and wellbeing in urban informal settlements: A scoping review in LMICs. Soc. Sci. Med..

[41] Lelijveld N., Benedict R.K., Wrottesley S.V., Bhutta Z.A., Borghi E., Cole T.J., Croft T., Frongillo E.A., Hayashi C., Namaste S. (2022). Towards standardised and valid anthropometric indicators of nutritional status in middle childhood and adolescence. Lancet Child Adolesc. Health.

[42] Mehare A., Abdisa L., Hawitibo A.L., Hundie S.K. (2026). The Food Price Changes and Gender Gap in Child Nutritional Status in Ethiopia. Child Indic. Res..

[43] Clark E.C., Reyce E., Neil-Sztramko S.E., Tarasuk V. (2025). Health outcomes following childhood or adolescent exposure to household food insecurity: A rapid systematic review. Public Health Nutr..

[44] Abraham S., Breeze P., Lambie-Mumford H., Sutton A. (2025). Household food insecurity and its impact on child and adolescent health outcomes in Western high-income countries: A rapid review of mechanisms and associations. Public Health Nutr..

[45] Frongillo E.A., Adebiyi V.O., Boncyk M. (2024). Meta-review of child and adolescent experiences and consequences of food insecurity. Glob. Food Secur..

[46] Rosen F., Settel L., Irvine F., Koselka E.P., Miller J.D., Young S.L. (2024). Associations between food insecurity and child and parental physical, nutritional, psychosocial and economic well-being globally during the first 1000 days: A scoping review. Matern. Child Nutr..

[47] St. Pierre C., Ver Ploeg M., Dietz W.H., Pryor S., Jakazi C.S., Layman E., Noymer D., Coughtrey-Davenport T., Sacheck J.M. (2022). Food insecurity and childhood obesity: A systematic review. Pediatrics.

[48] Kemper J.A., Kapetanaki A.B., Spotswood F., Roy R., Hassen H., Uzoigwe A.G., Fifita I.M. (2023). Food practices adaptation: Exploring the coping strategies of low-socioeconomic status families in times of disruption. Appetite.

[49] Mbhenyane X., Makuse S., Tambe A., Zuma M. (2025). Coping strategies for food insecurity among rural households: An observational study of adaptive mechanisms. Front. Sustain. Food Syst..

[50] Reytor-González C., Campuzano-Donoso M., Román-Galeano N.-M., Castano Jimenez J., Paz-Yépez C., Montalvan M., Simancas-Racines D. (2026). Socioeconomic disparities and nutritional aging: How food insecurity and financial hardship accelerate health decline in older adults. Front. Nutr..

[51] Borku A.W., Tora T.T., Masha M. (2025). Cassava in focus: A comprehensive literature review, its production, processing landscape, and multi-dimensional benefits to society. Food Chem. Adv..

[52] Al-Ansari M.A., Nabeel H., Abdella G.M., El Mekkawy T. (2025). Moving beyond indices: A systematic approach integrating food system performance and characteristics for comprehensive food security assessment. Foods.

[53] Tadesse L., Uncha A., Toma T. (2024). Multiple indicators-based assessment of rural food security status in landslide-prone areas of Southern Ethiopia. Discov. Sustain..

